# Magnetic fields induce exclusion zones in water

**DOI:** 10.1371/journal.pone.0268747

**Published:** 2022-05-27

**Authors:** Valery Shalatonin, Gerald H. Pollack

**Affiliations:** Department of Bioengineering, University of Washington, Seattle, Washington, United States of America; University of California, Merced, UNITED STATES

## Abstract

Hydrophilic materials immersed in aqueous solutions show near-surface zones that exclude suspended colloids and dissolved molecules. These exclusion zones (EZs) can extend for tens to hundreds of micrometers from hydrophilic surfaces and show physicochemical properties that differ from bulk water. Here we report that exposure of standard aqueous microsphere suspensions to static magnetic fields creates similar microsphere-free zones adjacent to magnetic poles. The EZs build next to both north and south poles; and they build whether the microspheres are of polystyrene or carboxylate composition. EZ formation is accompanied by ordered motions of microspheres, creating dense zones some distance from the magnetic poles and leaving microsphere-free zones adjacent to the magnet. EZ size was larger next to the north pole than the south pole. The difference was statistically significant when polystyrene microspheres were used, although not when carboxylate microspheres were used. In many ways, including both size and dynamics, these exclusion zones resemble those found earlier next to various hydrophilic surfaces. The ability to create EZs represents a feature of magnets not previously revealed.

## Introduction

Hydrophilic materials immersed in aqueous solutions show near-surface zones that exclude suspended colloids and dissolved molecules [1]. These exclusion zones (EZs) can extend for tens to hundreds of micrometers from hydrophilic surfaces and show physicochemical properties that differ from bulk water [1, 2].

Since water is the predominant component of most biological tissues, and has diamagnetic properties [3, 4], biological entities should be impacted by magnetic fields (MFs). Hence, any MF-induced changes in the physical and chemical properties of water could impact living organisms. Therefore, the interaction of MFs with water is of interest not only from a scientific perspective, but also in the context of applications in biology and medicine. Nevertheless, despite an abundance of experimental data, the mechanisms underlying magnetoreception have yet to be identified [3, 5].

Our experiments demonstrate a novel physical phenomenon occurring when water is exposed to MFs, opening a new possibility for explaining how magnetized water forms. Here we report that exposure of standard aqueous microsphere suspensions to static magnetic fields creates microsphere-free zones adjacent to magnetic poles, implying that magnetic fields build EZs.

## Materials and methods

### Sample preparation

The deionized (DI) water used in all experiments was obtained from a Barnstead D3750 Nanopure Diamond purification system (type-1 high-performance liquid chromatography grade, resistivity 18.2 MΩ·cm at 25°C). The DI water was passed through a 0.2-μm hollow-fiber filter, ensuring bacteria- and particle-free water. The pH of the resulting water was between 6.0 and 6.5.

A microsphere suspension in DI water was used for visualizing the EZ and determining its size. Two types of polymeric, non-magnetic microspheres were purchased from Polysciences, Inc.: Polybead polystyrene (PS), and Polybead carboxylate polystyrene (CPS), the latter containing surface carboxyl groups. Mean diameter of the microspheres was 2.0 μm, and bead density was ~1.05 g/cm^3^. The microspheres are packaged as 2.5% solids (weight/volume) aqueous suspensions containing 5.68 x 10^9^ particles/mL. All experiments were carried out using mixtures of the microsphere suspension with DI water at a ratio of 45 μL (1 drop)/25 mL. Suspension homogeneity was achieved by means of hand or mechanical agitation. Experiments were carried out at room temperature (296°K < T < 299°K).

### Experimental setup

**Fig 1:**
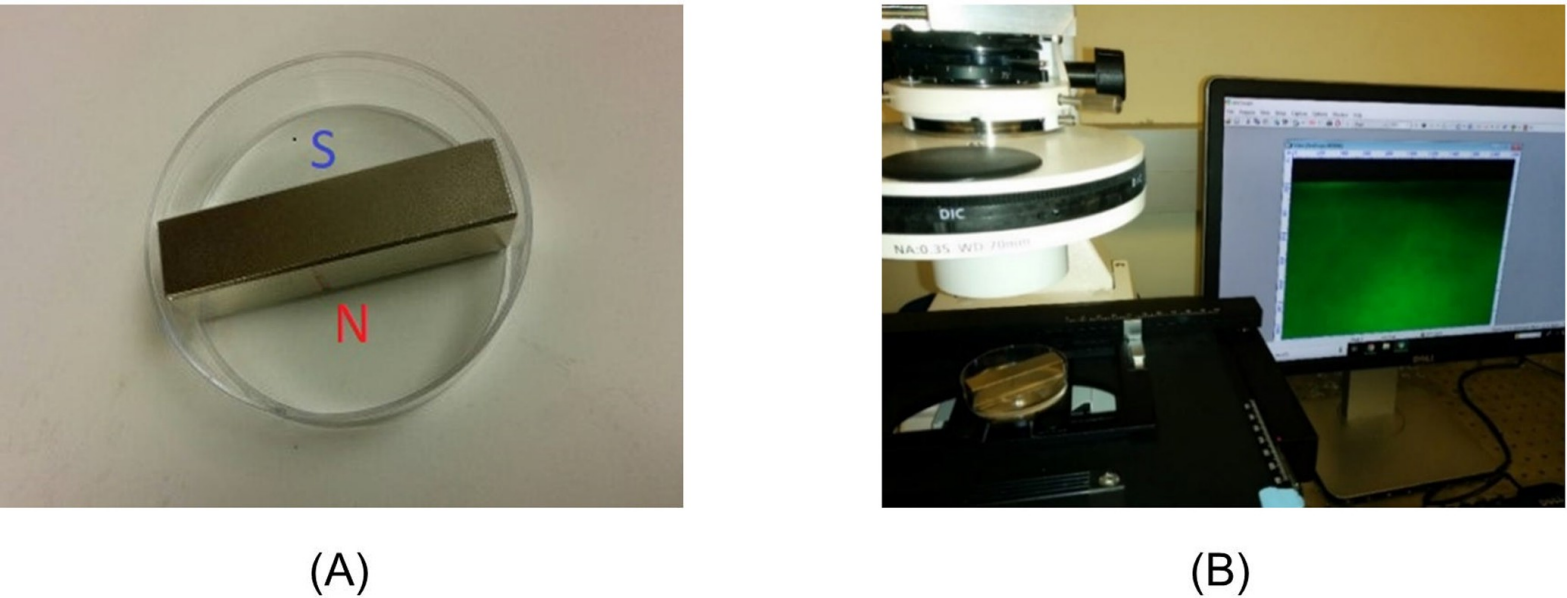
Setup 1 for studying EZ formation. (A) Magnet placed inside of a water-filled petri dish. (B) Microscopic setup for observing EZ formation. The magnets and Petri dishes images are for illustrative purposes only.

In the first setup (Setup 1), we used uncovered sterile polystyrene petri dishes (60 x 15 mm, cat. No. AS4052, Termo Fisher Scientific) containing 4 mL of the microsphere suspension. A rectangular magnet 50 x 7 x 7 mm (2" x 1/4" x 1/4") was positioned in the middle of the petri dish, so that ~2 mL of the microsphere suspension remained on each side of the magnet (Fig 1A). The height of the water suspension was 2–3 mm. The neodymium bar magnet, with nickel-plated surface, was purchased from CMS Magnetics, Inc. (SKU: NB021424-52N). The field strength near both poles of the magnet was 1.44 Tesla. To observe EZ formation, the setup was placed on the stage of a Zeiss Axiovert 35 inverted microscope (Fig 1B). Inverted microscope stages do not translate up and down. Focusing is accomplished by using a translatable nosepiece beneath the stage, which, together with the objectives, moves up and down.

**Fig 2:**
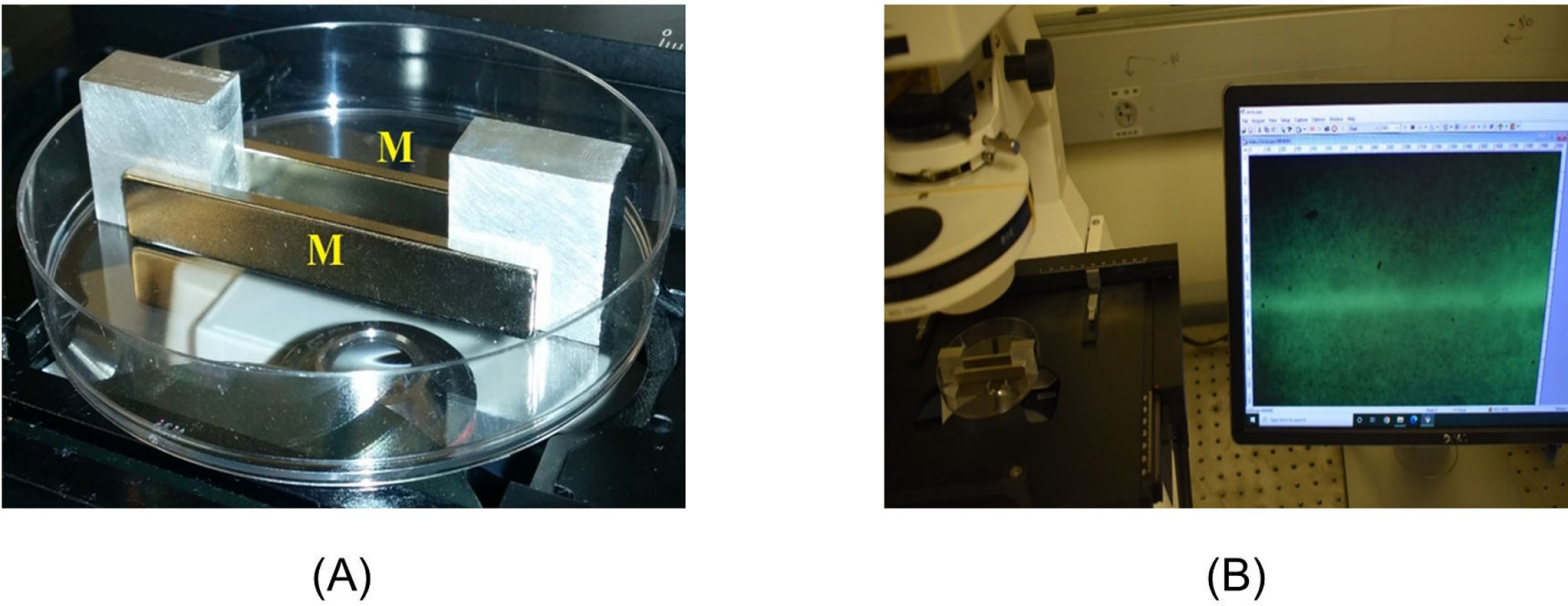
Setup 2 for studying the EZ formation. (A) Two parallel magnets (M), separated by aluminum blocks, placed inside of a petri dish. (B) Microscopic setup for observing EZ formation. The magnets and Petri dishes images are for illustrative purposes only.

Therefore, the vertical position of the focus, and consequently the height of the in-focus microspheres from the petri dish bottom, could be adjusted by lifting and lowering the objective relative to the microscope stage. The microscope was fitted with a 5X objective (Zeiss, N.A. = 0.13) and a 10X objective (Zeiss, N.A. = 0.25). All microscope images were obtained using the 5X lens. The 10X lens was used additionally for observing microsphere movement. We used a video camera (Am Scope MD 800E) for capturing color images and recording videos. Setup 2 was used to study EZ formation in a more uniform MF (Fig 2). Two parallel, separated, neodymium bar magnets (76 x 12 x 3 mm) were placed inside a sterile polystyrene petri dish (95 x 15 mm), Termo Fisher Scientific). The magnets were purchased from CMS Magnetics, Inc. The field strength near both poles of the magnet was 1.35 Tesla.

The distance between the magnets was set at 8 mm using aluminum (non-magnetic) block separators (Fig 2A). North-south attraction kept the magnets securely in place. To conduct experiments, we used 1.5–2 mL of the freshly prepared CPS or PS microsphere suspensions. The suspension was poured into the petri dish between the magnets. The petri dish with the magnets was placed on a microscope stage (Fig 2B) for observation. Experiments were conducted at room temperature (296°K < T < 299°K).

### Statistical analysis

The data analysis software, Tool Pak, installed in Microsoft Excel, was applied to perform statistical analysis. A two-sample t-Test, assuming equal variances and comparing the means of two samples, was used. We adopted a significance level (threshold of significance) α = 0.05. A p-value less than 0.05 was then taken as statistically significant. This value is a measure of the probability that an observed difference could have occurred by random chance.

## Results

**Fig 3:**
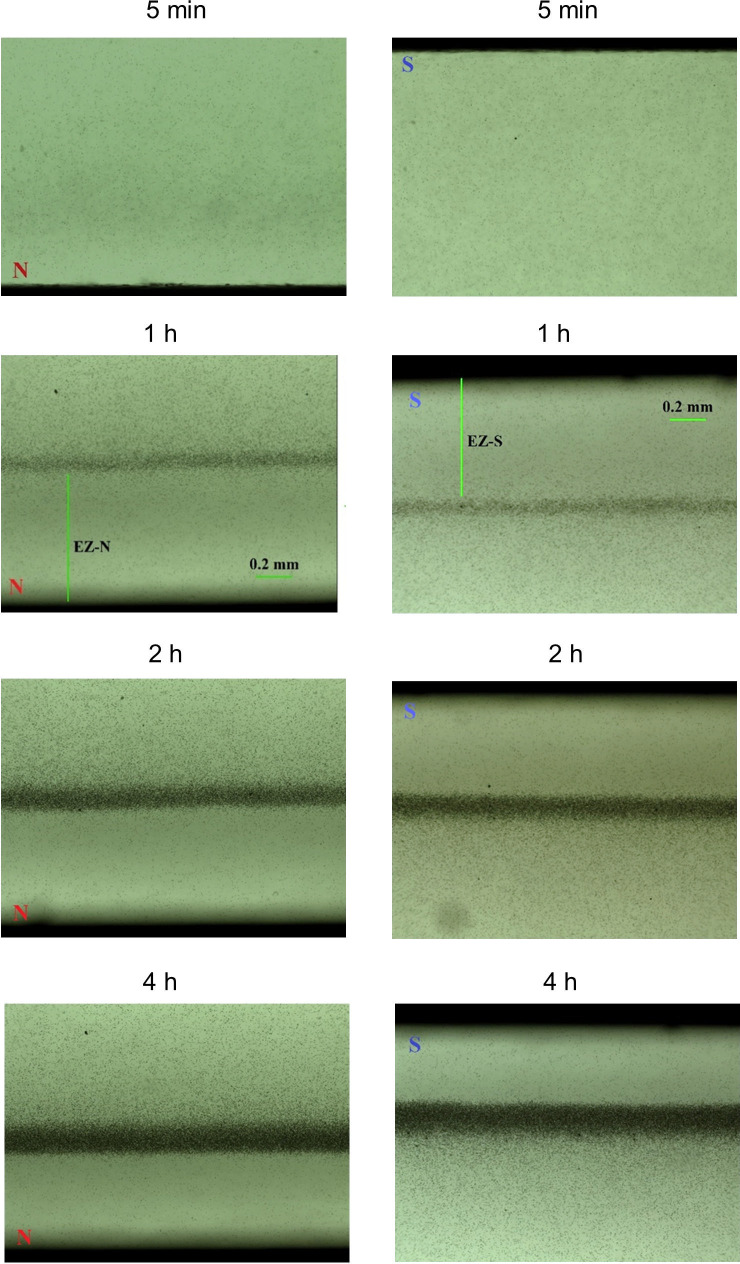
Representative time-dependent changes in microsphere concentration near North (N) and South (S) poles.

Experiments carried out using Setup 1 showed that EZs were formed adjacent to both the magnet’s N and S surfaces (Fig 3). Changes in disposition of the suspended microspheres arose immediately after the start of the experiment. After 20–30 minutes, a reduced concentration of microspheres could be observed near the magnet, along with a layer with an increased concentration beyond that zone—as though the microspheres had migrated away from the zone near the magnet and concentrated in that more-distant zone. We refer to the microsphere-depleted zone as the exclusion zone, or “EZ.”

Over the longer term, the EZ was sustained, but not fully. After the microsphere-rich layer consolidated, the distance between the magnet and dense layer gradually diminished, even as the number of microspheres between the layer and the magnet decreased, to form increasingly distinct EZs. This occurred at both the north and south poles.

We checked whether the formation of EZ might arise from the metallic surface of the magnet rather than from the magnet’s field. To test this, we completely enveloped the magnet with a thin (70 μm) polyethylene film. Results were indistinguishable from those obtained without the film.

Many phenomena related to the magnetic treatment of water may involve the environment. For example, the magnetization of pure water appears to require dissolved oxygen or air [6]. To check for the possible influence of the environment, the microsphere suspension was covered by a glass slide, isolating it from the environment. A slow decrease of microsphere velocity was observed, beginning 20–25 seconds after covering, and motion nearly ceased by two to three minutes except for modest vibrational oscillations. Further EZ growth also stopped. Only after removing the glass slide did the collective movement of the microspheres gradually restart. S1 and S2 Videos show typical changes in microsphere movement.

Plotting EZ growth vs. time met with some obstacles. Although microsphere movements began immediately after the microsphere suspension was poured into the Petri dish, the EZ was not well enough defined early on to be measurable with confidence; hence, values were plotted beginning at 30 minutes (Fig 3). CPS microspheres were used.

By 60 minutes, the EZ was more discernible, and by two hours the EZ became rather clear, because microspheres were more substantially excluded (Fig 3). Some microspheres remaining in the EZ had settled to the bottom of the Petri dish, or were still moving away from the magnet while at the same time descending to the bottom of the Petri dish. Progressive formation of EZs next to both poles can be seen. Later, we found a gradual decrease of EZ size, whose magnitude differed for regions adjacent to north and south poles (see below).

**Fig 4:**
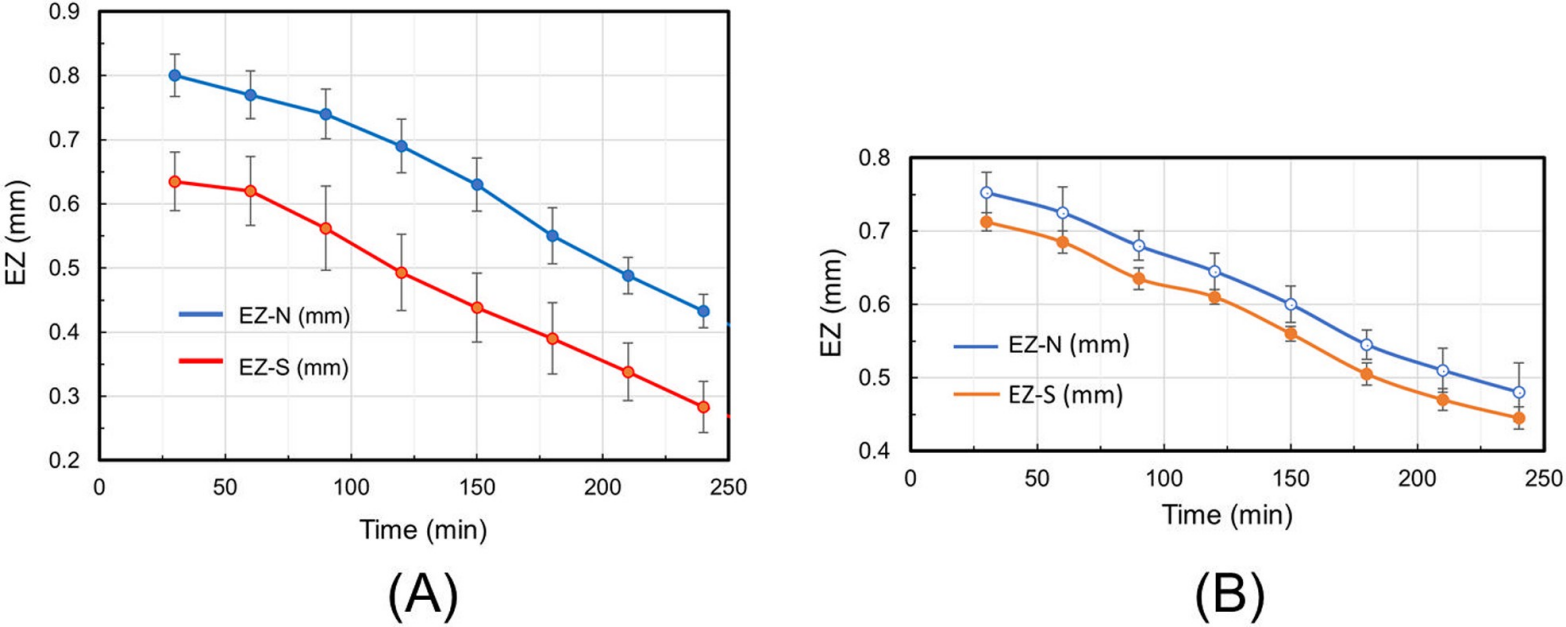
The time course of EZ size: A—PS microspheres; B—CPS microspheres. Error bars denote standard deviations. The differences between the averaged curves are statistically significant for PS microspheres (*p* = 0.012) and not statistically significant (*p* = 0.22) for CPS microspheres. The number of experiments, *n* = 3 (for PS and CPS microspheres).

**Fig 5:**
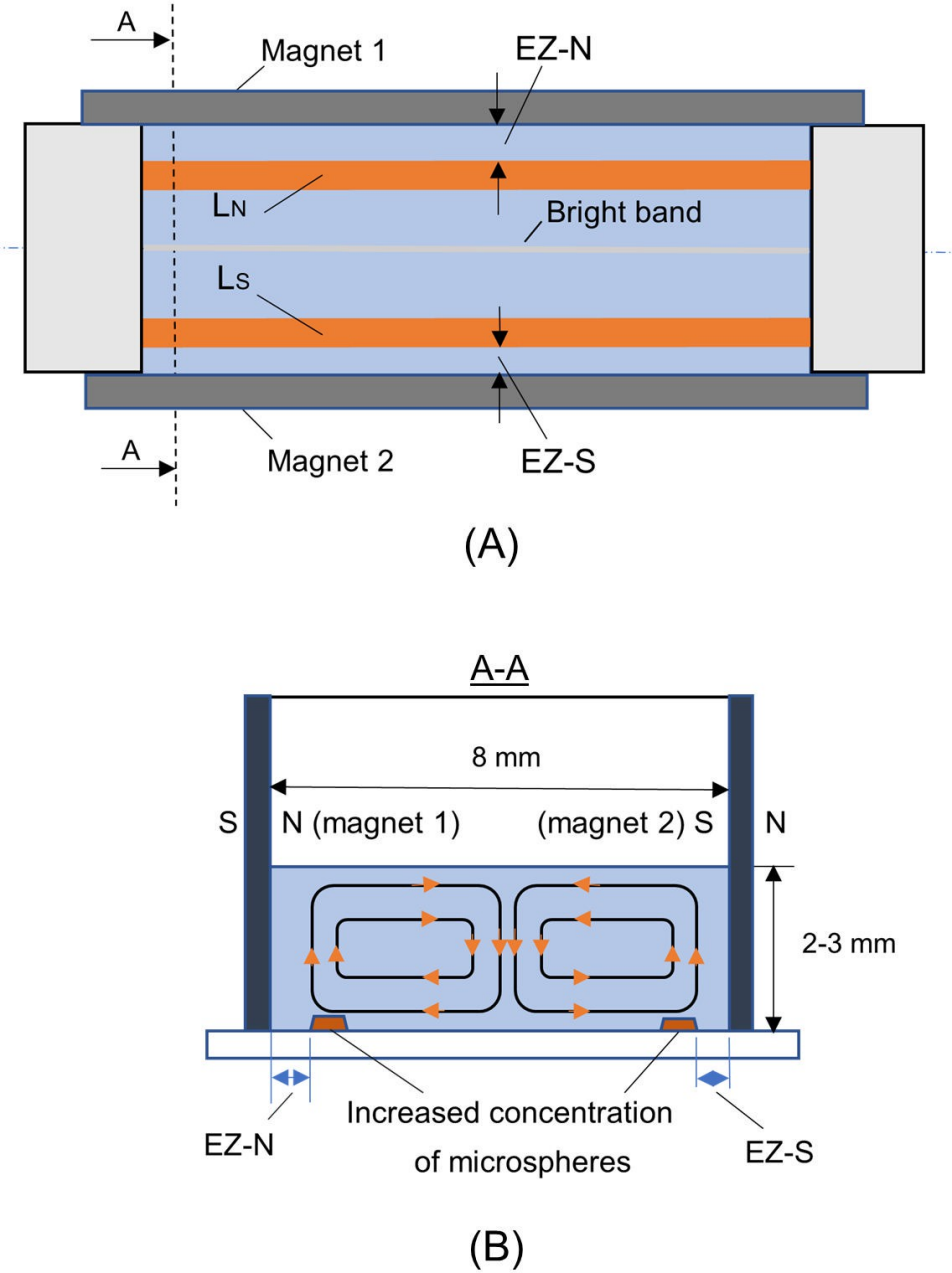
Schematic drawing of experimental Setup 2. (A) Exclusion zones, EZ-N and EZ-S formed near poles of magnets 1 and 2. L_N_, L_S_==layers of increased microsphere concentration near North (N) and South (S) poles. (B) Oval closed lines show the patterns of movement of microspheres. A bright microsphere-free band (width 30–200 μm), which is located halfway between the magnets, is readily visible under the microscope at all depths of the water suspension.

Fig 4A and 4B show the time course of EZ size measured using polystyrene and carboxylate polystyrene microspheres, respectively. Each plot is an average of data from three independent experiments (S1 Data set). EZ size is defined as the extent of the microsphere-free region. When using polystyrene microspheres, EZ size next to the N pole was 15–20% larger than the same values for the S pole (Fig 4A). The difference between the averaged curves was statistically significant (*p* = 0.012). This result is illuminating because it suggests physical differences in the properties of N and S poles. Researchers often pay little attention to MF polarity, i.e., to whether the surface of the magnet nearest the object of interest is the north (N) or south (S) pole, presuming that the field, and its impacts are similar. Fig 4A implies that that presumption might not be valid. When using CPS microspheres, however, the difference between N- and S- EZs was smaller (Fig 5), and the difference was not statistically significant (*p* = 0.22).

Most measurements were repeated using Setup 2, which contained two magnets, with a uniform MF in between (Fig 2). Such arrangement helped provide new and essential information on microsphere motions during EZ buildup. Motion between the magnets arose immediately after the start of the experiment and involved nearly all microspheres. By adjusting the vertical focal plane of the microscope lens, we could observe this motion at different depths of the microsphere suspension.

The main features of these motions are summarized in Fig 5. Panel A shows a schematic view from above the setup. Panel B depicts the microsphere movements, which were nearly symmetrical about the horizontal axis. Surprisingly, microspheres moved along closed oval trajectories. While moving, they gradually got excluded from S- and N- pole regions, forming the EZs as well as the layers L_N_, L_S_ of increased concentration at some distance from both poles (Fig 3). The size and form of the EZ are similar to those obtained in Setup 1. The size similarity is not surprising because in both cases EZ formation occurs in close vicinity of the magnetic poles, where the magnetic field lines differ little between Setup 1 and Setup 2.

**Fig 6:**
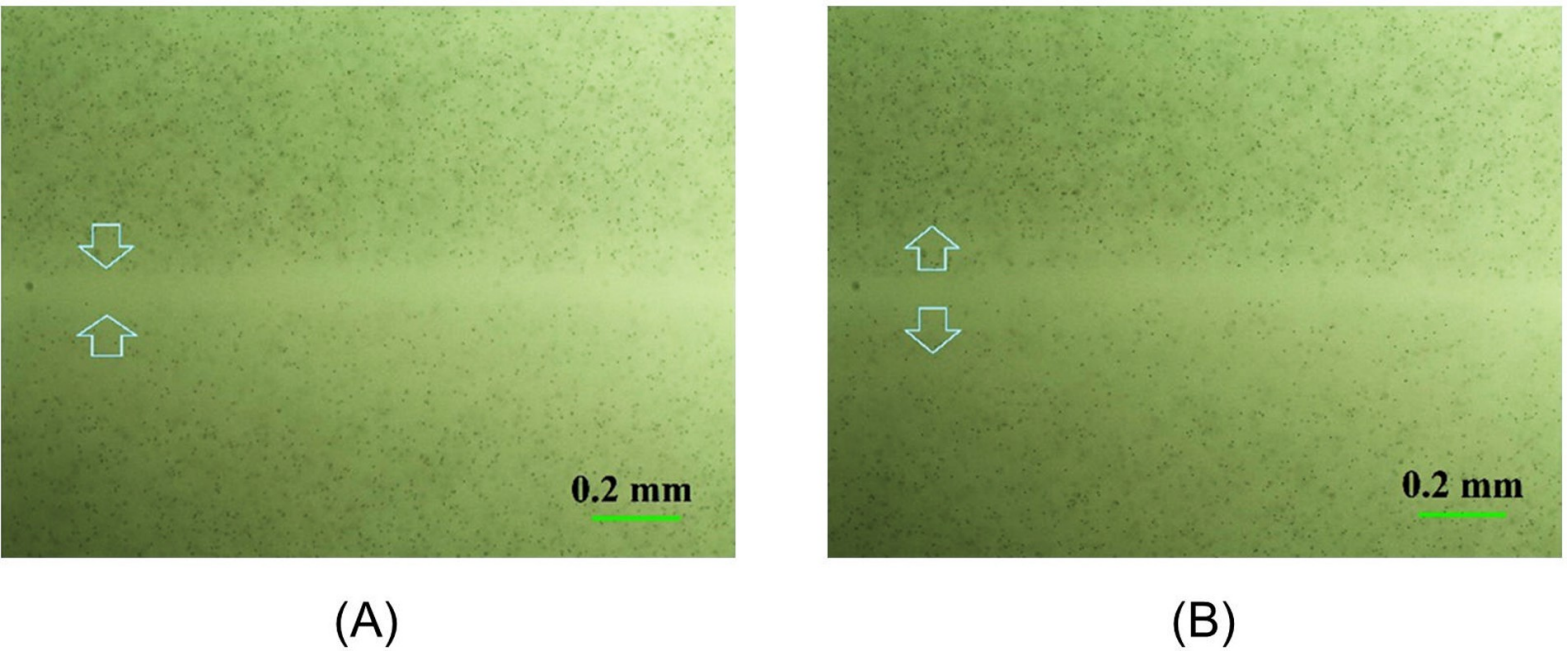
Microsphere-free band forms between magnets 1 and 2. (A) near the surface, (B) near the bottom of the Petri dish. The arrows show the directions of microsphere motion at the respective levels.

Another characteristic feature of microsphere dynamics in Setup 2 is shown in Fig 6. When the microscope lens was focused at the midpoint of the water-microsphere suspension, at any height, a bright microsphere-free band was observed (Figs 5A and 6). PS and CPC microspheres were used. Such bands were seen in all of the more than ten experiments carried out with Setup 2. The width of the band diminished somewhat when the focus was shifted to near the bottom of the chamber (Figs 6B and S1 and S2). Possibly, the absence of microspheres in that band may have arisen from Coulomb forces. But overall, even taking into account the known electrical and magnetic properties of microspheres, the reasons for all of the observed features remain unclear and need to be studied in more detail.

The dynamics of microsphere motion near the bright band of Fig 6 can be seen in S3 and S4 Videos. The microscope lens was focused on the microspheres situated near the suspension surface (S3 Video) or near the bottom of the Petri dish (S4 Video). Thus, S3 and S4 Videos relate to Fig 6A and 6B, respectively. Although those videos began at about 30 min after the experiment had started, the ordered microsphere motion arose immediately after the start of the experiment. The directions of microsphere motion coincided with the arrow directions in Fig 6. In turn, this corresponds to the direction of their motion shown in Fig 5B (viewed from below). Microsphere dynamics in S3 Video (near the surface) appear to be more unstable than in S4 Video (near the bottom). The microspheres also had a fairly sizable lateral motion component, possibly, explainable by the influence of the environment.

## Discussion

The main finding in this study is that microsphere-free zones build next to magnets immersed in aqueous suspension. They build next to both north and south poles; and they build whether the microspheres are polystyrene (PS) or carboxylate (CPS). Hence microsphere-free zones appear to be characteristic features of magnets immersed in aqueous suspensions.

### Mechanism

A question that immediately arises is whether the exclusion zones arise directly from the magnetic fields themselves, or from features of the water exposed to those magnetic fields. The former seems unlikely: Charged objects generally move perpendicular to the magnetic field lines, not parallel as in the current experiments (Fig 5B). Hence, the movement did not appear to arise from the direct force of the magnetic field on the microspheres.

The alternative interpretation is that the exclusion zones (EZ) arise from an impact of the magnet on nearby water molecules. The magnet’s material, itself, does not appear to have been responsible, for enveloping the magnet with a thin polyethylene film produced indistinguishable results. Yet, some feature of the magnetic field created EZs resembling those previously observed next to many surfaces [1]. The size observed here, <1 mm, is typical, and so is, roughly, the time course–building to full extent within less than tens of minutes. On the other hand, other reported features of EZs were not explored.

Hence, good reason exists to suggest that the EZs seen here bear similarity to the EZs seen next to many other materials. Those EZs appear to result from a transition of water molecules from their ordinary random arrangement to a quasi-crystalline arrangement which is denser than water, and which excludes numerous particles and molecules (hence the nomenclature, “exclusion” zone).

Another similarity is the energy required to drive the microsphere motion. In the case of the standard EZ, infrared has been established as the main driving energy [2]. In those studies, adding infrared energy created larger EZs, while blocking infrared resulted in diminution. In the current experiments we blocked infrared energy by positioning a glass slide above the chamber, and found that the microsphere movement that created the EZ ceased. Hence, Infrared may constitute the energy driving the phenomenon, as is the case with usual EZs [1].

Why, then do microspheres move away from the magnet’s surface, creating those EZs? In the case of standard EZs, some features of the material-surface charges seem to bear responsibility. In the case of magnets, we don’t ordinarily think of surface charges or the electric fields they create, but the present results raise question whether magnets could contain surface charges similar to those of many ordinary materials [7, 8].

### Dynamic features

Several additional features of the results warrant discussion. The first is the broad, dark band that forms at the far edge of the exclusion zone. Once again, this is a common feature of exclusion zones [1]. EZs form as water molecules split; the negative, OH^-^ moiety forms the EZ, while the H^+^ moiety gets excluded, beyond the region of the EZ. Those H^+^ (protons) tend to link the negatively charged microspheres, forming dense zones, similar to those observed here. Hence the presence of those dark regions are unsurprising.

A second feature is the clear zone that developed in the two-magnet configuration (Setup 2). The band was situated halfway between the two magnets (Figs 5A and 6), and it was seen consistently (S1 Fig). We presume this band arose because of electrostatic repulsion between the microspheres, although another possibility is the formation of yet another EZ, created by microsphere surfaces on either side of the clear zone.

A third feature is the difference of size of EZs next to north vs. south poles. With carboxylate (CPS) microspheres, the difference was not statistically significant; but with polystyrene (PS) microspheres, there was a statistically significant difference, on the order of 15%, the north being larger than the south. That difference may be related to the microspheres’ interactions with water. Polystyrene microspheres have hydrophobic surfaces, with only slight negative charge from sulfate esters [9]. Carboxylate microspheres are also negatively charged but contain hydrophilic surface-carboxyl groups (COOH). Thus, carboxylate microspheres may well interact with vicinal water, possibly explaining the quantitative differences between the two microsphere types.

If the EZs result from surface charges on the magent’s surface, then, possibly, the charges on the north pole are more effective in nucleating EZ growth. The recognized sensitivity of different processes to the polarity of the magnetic field may well be relevant here [10, 11].

### Biological context

Magnetic fields impact biological organisms; yet the mechanisms by which this occurs have not yet been pinpointed [4, 5]. The long-range effects observed here could play a role in understanding of these mechanisms. If the magnets separate charges in the same manner as occur with standard EZs [1], then the same mechanism may apply biologically. With separated charges, biological effects of one sort or another would seem inevitable. This seems an area worthy of further exploration.

Indeed, much of the water in living cells lies very near to one or another hydrophilic surface, and as that water is interfacial, it should likely be ordered (EZ). If so, then ordered water needs to be considered when considering cell behavior. The fact that EZ water can be created by static magnetic fields is therefore significant: it provides new ways to build EZ water, and by so doing, may help explain the well-known therapeutic properties of magnetic fields. In this context, particularly interesting is the observation that the influence of N water and S water may differ. Various studies indeed show that these respective waters exhibit different therapeutic features [12–14]. The emerging questions are: how? and why? These issues will need further study.

In conclusion, an unexpected feature of magnets has been uncovered: the presence of robust microsphere-exclusion zones next to north and south poles. In many ways these exclusion zones resemble those found earlier next to various hydrophilic surfaces. They imply features of magnets not previously revealed.

## Supporting information

S1 Video Influence of the environment on microsphere movement. A Petri dish with a PS-microsphere suspension and a magnet were placed on the microscope stage for examination. A 5X objective was used. The microscope was focused on the PS microspheres situated in the upper part of the suspension, near the N pole side of the magnet. In the inverted microscope the objectives are located below the Petri dish. That arrangement compromised the ability to obtain a well-focused magnet edge. The dark blurred line in the video, seen just below the label, is the N-pole edge of the magnet, which is in contact with the microsphere suspension. We observed the usual movement of microspheres at first. Then, a glass slide was installed 11–12 seconds following the start of the video recording (15 minutes after the experiment began). A slow decrease of microsphere motion began within approximately 20–25 seconds, and by the end of the video (100 seconds later) their motion became very slow.
(MP4)

S2 Video Influence of the environment on microsphere movement. This video is closely connected with the S1 Video. Here, the microscope objective was once again focused on the microspheres situated at the upper part of the suspension, but near the S pole instead of the N pole. Microsphere movement had practically stopped by ~130 seconds. The glass slide was then removed. The video recording had started 13 seconds before removing the glass slide. Apparently, due to the presence of the glass slide the microspheres practically did not move except for slow oscillations relative to middle positions. After removing the glass slide (13–18 seconds), the ordered movement of microspheres gradually restarted. By 40–45 seconds, their motion became clearly visible, and their velocity gradually increased. By the end of the video, the dynamics and velocity of microsphere movement became more stable.
(MP4)

S3 Video Microsphere motion in the region of the bright band, near the surface of the suspension. It is seen that microspheres move towards each other in the direction of the microsphere-free zone (bright band). There is a quite big longitudinal component in their motion (parallel to the bright band). The closeness of the environment (interaction with the absorbed gases) possibly effects on the stability of the microspheres motion. The absence of microspheres in the bright band may be explained by Coulomb forces arisen between charged microspheres. The observed motion is in accordance with Fig 5B.
(MP4)

S4 Video Microsphere motion in the region of the bright band, near the bottom of the petri dish. The observed motion is in accordance with Fig 5B (near bottom). The microspheres move away from each other towards the N and S poles of magnets 1 and 2. Their motion is more s`than in the upper part of the suspension layer (S3 Video). The observed microsphere-free zone is the area between two closed oval motions of microspheres (Fig 5).
(MP4)

S1 Fig Bright microsphere-free band. The microscope lens was focused on the microspheres situated in the upper part of the *s*uspension of polystyrene microspheres (2 μm diameter) in pure deionized water. The usage of a green light filter increased the contrast of details of the photo.
(TIF)

S2 Fig Bright microsphere-free band. The microscope lens was shifted to near the bottom of the Petri dish. The width of the band diminished somewhat. The usage of a green light filter increased the contrast of details of the photo.
(TIF)

S1 Data set (DOCX)
