# Supplementary figures and images for: Magnetic fields induce exclusion zones in water
Source: PLoS One. 2022 May 27;17(5):e0268747. doi: 10.1371/journal.pone.0268747 (PMC9140229; doi:10.1371/journal.pone.0268747)

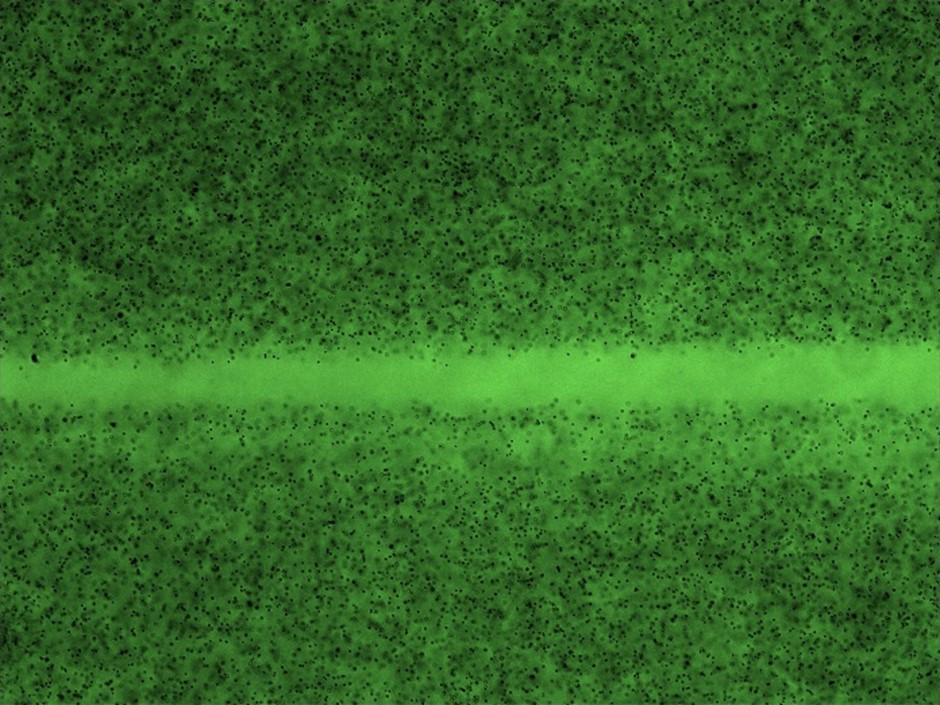

Supplement: S1 Fig — The microscope lens was focused on the microspheres situated in the upper part of the suspension of polystyrene microspheres (2 μm diameter) in pure deionized water. The usage of a green light filter increased the contrast of details of the photo. (TIF) [file pone.0268747.s005.tif]

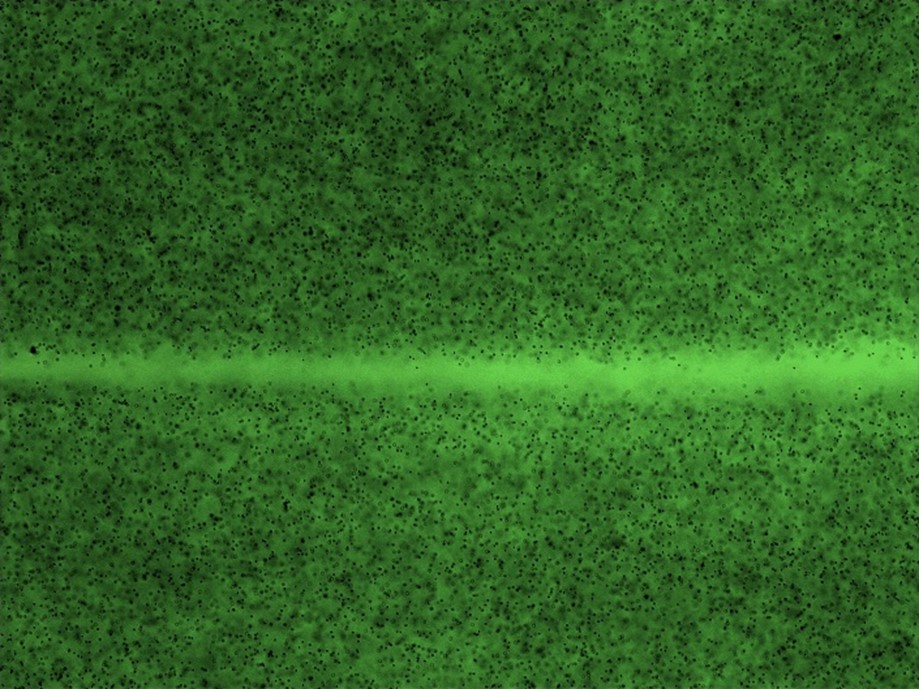

Supplement: S2 Fig — The microscope lens was shifted to near the bottom of the Petri dish. The width of the band diminished somewhat. The usage of a green light filter increased the contrast of details of the photo. (TIF) [file pone.0268747.s006.tif]
