# Supplementary material for: Magnetic fields induce exclusion zones in water
Source: PLoS One. 2022 May 27;17(5):e0268747. doi: 10.1371/journal.pone.0268747 (PMC9140229; doi:10.1371/journal.pone.0268747)
Supplement: S1 Data set — (DOCX) [file pone.0268747.s007.docx]

**Supporting information**

**S1 Tables A and B. Data which were used to construct Fig 4.**

Time courses of EZs formed in the vicinity of N (S1 Table A) and S (S1 Table B) poles of a magnet were measured. Three experiments were carried out. Polystyrene (PS) microspheres suspended in DI water were used.

**S1 Table A**

| **Time (min)** | **EZ-N1 (mm)** | **EZ-N2 (mm)** | **EZ-N3 (mm)** |
| --- | --- | --- | --- |
| **30** | 0.81 | 0.84 | 0.75 |
| **60** | 0.78 | 0.81 | 0.73 |
| **90** | 0.75 | 0.78 | 0.69 |
| **120** | 0.67 | 0.74 | 0.65 |
| **150** | 0.64 | 0.67 | 0.57 |
| **180** | 0.54 | 0.6 | 0.5 |
| **210** | 0.5 | 0.54 | 0.43 |
| **240** | 0.43 | 0.47 | 0.4 |

**S1 Table B**

| **Time (min)** | **EZ-S1 (mm)** | **EZ-S2 (mm)** | **EZ-S3 (mm)** |
| --- | --- | --- | --- |
| **30** | 0.7 | 0.58 | 0.62 |
| **60** | 0.67 | 0.56 | 0.63 |
| **90** | 0.62 | 0.49 | 0.56 |
| **120** | 0.57 | 0.41 | 0.5 |
| **150** | 0.50 | 0.36 | 0.45 |
| **180** | 0.45 | 0.32 | 0.4 |
| **210** | 0.4 | 0.26 | 0.35 |
| **240** | 0.34 | 0.23 | 0.28 |

**S1 Tables C and D. Data which were used to construct Fig 5.**

Time courses of EZs formed in the vicinity of N (S1 Table C) and S (S1 Table D) poles of a magnet were measured. Three experiments were carried out. Carboxylate polystyrene (PS) microspheres suspended in DI water were used.

**S1 Table C**

| **Time (min)** | **EZ-N1 (mm)** | **EZ-N2 (mm)** | **EZ-N3 (mm)** |
| --- | --- | --- | --- |
| **30** | 0.73 | 0.78 | 0.74 |
| **60** | 0.69 | 0.76 | 0.72 |
| **90** | 0.66 | 0.7 | 0.65 |
| **120** | 0.62 | 0.67 | 0.66 |
| **150** | 0.56 | 0.61 | 0.6 |
| **180** | 0.53 | 0.56 | 0.54 |
| **210** | 0.49 | 0.55 | 0.5 |
| **240** | 0.44 | 0.52 | 0.48 |

**S1 Table D**

| **Time (min)** | **EZ-S1 (mm)** | **EZ-S2 (mm)** | **EZ-S3 (mm)** |
| --- | --- | --- | --- |
| **30** | 0.7 | 0.73 | 0.71 |
| **60** | 0.67 | 0.7 | 0.68 |
| **90** | 0.62 | 0.65 | 0.64 |
| **120** | 0.6 | 0.62 | 0.63 |
| **150** | 0.57 | 0.55 | 0.57 |
| **180** | 0.56 | 0.53 | 0.55 |
| **210** | 0.52 | 0.49 | 0.51 |
| **240** | 0.43 | 0.46 | 0.45 |
